# Olive-Fruit Mass and Size Estimation Using Image Analysis and Feature Modeling

**DOI:** 10.3390/s18092930

**Published:** 2018-09-03

**Authors:** Juan Manuel Ponce, Arturo Aquino, Borja Millán, José Manuel Andújar

**Affiliations:** University of Huelva, Department of Electronic Engineering, Computer Systems and Automation, La Rábida, Palos de la Frontera, 21819 Huelva, Spain; arturo.aquino@diesia.uhu.es (A.A.); borja.millan@diesia.uhu.es (B.M.); andujar@diesia.uhu.es (J.M.A.)

**Keywords:** olive, food industry, fruit grading, image analysis, segmentation

## Abstract

This paper presents a new methodology for the estimation of olive-fruit mass and size, characterized by its major and minor axis length, by using image analysis techniques. First, different sets of olives from the varieties Picual and Arbequina were photographed in the laboratory. An original algorithm based on mathematical morphology and statistical thresholding was developed for segmenting the acquired images. The estimation models for the three targeted features, specifically for each variety, were established by linearly correlating the information extracted from the segmentations to objective reference measurement. The performance of the models was evaluated on external validation sets, giving relative errors of 0.86% for the major axis, 0.09% for the minor axis and 0.78% for mass in the case of the Arbequina variety; analogously, relative errors of 0.03%, 0.29% and 2.39% were annotated for Picual. Additionally, global feature estimation models, applicable to both varieties, were also tried, providing comparable or even better performance than the variety-specific ones. Attending to the achieved accuracy, it can be concluded that the proposed method represents a first step in the development of a low-cost, automated and non-invasive system for olive-fruit characterization in industrial processing chains.

## 1. Introduction

Olive growing is a high relevance agricultural activity. With a huge presence in the Mediterranean Basin, where its importance transcends the farming scope to become an actual symbol of its culture and tradition, the olive crop has spread all over the world [1,2]. Because of the well-proved health benefits of olive-derived products, and the excellence of its culinary uses, its consumption has considerably risen in recent years. According to IOC (International Olive Council) [3], table olives consumption has been increased by 173% in the twenty-five years between 1990/91 and 2015/16. Moreover, according to IOC and USDA (United States Department of Agriculture) expectations [3,4], olive oil consumption will exceed 3,000,000 tons in 2017/18.

To meet such demand, the olive industry must face multiple challenges. Despite the numbers of its market, olive farming and processing are still mainly performed in a traditional way. Even in Spain, the world largest producer, olive farming is still strongly linked to traditional production systems and low-density olive groves [5]. This model represents a problem in terms of productivity and profitability. In recent years, super-high-density olive groves, along with increased mechanization, have been introduced as response. Although some indicators suggest that these solutions, based on intensification, can provide the key for economic survival, accurate knowledge about its impact and viability is still yet to be obtained [6]. Be that as it may, within this context, the enhancement and modernization of the processes, and the introduction of innovative solutions at all levels, are fundamental tasks to be accomplished by this industry.

Fruit sizing is a high-relevance post-harvest task in the food industry [7]. Sorting fruits and vegetables according to different attributes such as color, mass, size or shape can all be determinants in the quality and pricing of the eventual product. In the olive sector, this is especially relevant [8,9]. For table olives, the uniform size, spotless surface or appropriate coloring are determining quality-features as perceived by the final consumer. On the other hand, focusing on olive oil, size and mass of the fruits are used to calculate yield estimations. In any case, the measurement of these parameters is a necessity. However, measuring the whole harvested batch by hand is not an option due to the huge workload involved. Thus, the actual processes to extract this information are based on the study of samples.

In recent years, machine vision techniques have been explored as a valuable tool in food industry. Within the precision agriculture and the horticultural product manufacturing scopes, there is considerable literature regarding the use of image analysis to approach different problems, such as yield estimation, fruit detection, data extraction, sorting and classification or sizing and grading. Thus, Aquino et al. [10] presented a classification-based algorithm to predict grape yield at early stages from images taken on-the-go directly in the vineyard. Mery et al. [11] proposed a methodology for the detection, via image segmentation, of different kinds of food previously photographed. Cervantes et al. [12] developed a comparative analysis of different methods of feature extraction and classification of plant leaves using image processing techniques. Zhang et al. [13] developed an automatic fruit recognition system based on a split-and-merge algorithm and multiclass support vector machine (SVM). Sa’ad et al. [14] estimated the mass of mangoes from different photographs of the fruits using thresholding segmentation and provided a classification methodology supported by the information extracted from the results of this segmentation. In the same vein, Mizushima et al. [15] proposed a method for sorting and grading apples, based on the Otsu’s method and linear SVM. Omid et al. [16] performed the estimation of volume and mass of citrus fruits through the segmentation of images captured in the laboratory.

Regarding the olive sector, literature focused on the application of machine vision for olive treatment and manufacturing can be consulted. Of special relevance are developments for the classification of fruits according to different characteristics, such as defects on the surface [17] or the variety [18,19], and fruit detection for feature estimation [20].

This paper proposes an efficient methodology to estimate the maximum/minimum (polar/equatorial) diameter length and mass of olive fruits by means of image analysis. To this end, as a first step, the contrast between the olives and the background is maximized in the images by employing specialized morphological processing. Then, the olives are segmented by automated thresholding based on statistical bimodal analysis. Finally, estimation models for the targeted features are obtained by correlating measurements taken from the segmentations to actual values measured in the laboratory.

The manuscript is structured as follows: Throughout three subsections, Section 2 describes the experimental design and the data acquisition process, the developed image analysis algorithm and model training for olive characterization. The next section presents the methodology proposed for result evaluation and discusses the achieved results; they have been placed together in order to provide the best understanding of the paper’s research. Finally, the manuscript ends with the main conclusions on the carried-out research.

## 2. Materials and Methods

### 2.1. Reference Data and Image Acquisition

Olive fruits from two different varieties were considered for this study: Arbequina and Picual. Samples of both varieties were manually collected in January 2018, in high-density olive orchards located in Lagar Oliveira da Serra (Ferreria do Alentejo, Portugal).

Two populations (one per variety) of 200 olive fruits were selected from the samples previously acquired. Then, they were separated into different groups. Hence, for the Arbequina variety, the following sets were established: A1 (40 fruits), A2 (40 fruits), A3 (40 fruits), A4 (50 fruits) and A5 (30 fruits). For the Picual variety, four groups of 50 olives each were set up and were named as follows: P1, P2, P3 and P4.

**Figure 1:**
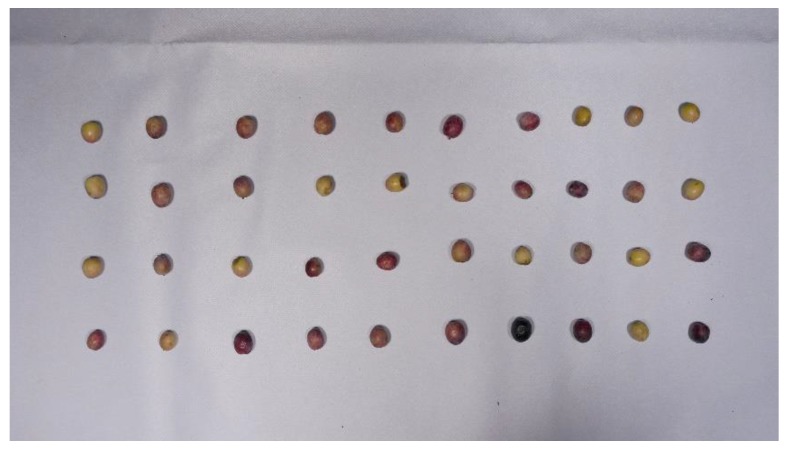
Example of image captured of the A1 set.

Every described set was photographed in the laboratory, spatially distributing olives over a white plastic mat. This durable and deformable material was chosen in an attempt to approximate the type that would be used in a real conveyor belt. For capturing, the LUMIX DMC-GH4 digital single-lens mirrorless camera, equipped with a NMOS sensor, was used (Panasonic, Kadoma, Osaka, Japan). It was set up in manual mode, with an aperture of f/8, an exposure time of 1/500 s, an ISO value of 400 and a focal length of 14 mm. To reproduce an environment close to an actual industrial system, an artificial lightning setup composed of two 500 W halogen floodlights, with a light appearance of 3300 k, was employed for scene illumination. The camera was perpendicularly located above the scene; the lights were placed at the same plane and oriented to the point the camera was focused on. Figure 1 shows an example of the captured images, which were acquired and saved in JPG format, with 4608×2592 pixels in resolution, a pixel density of 180 ppi and a color depth of 24 bits.

To evaluate the error produced by the estimation models, objective measurements of the major and minor axis length (in millimetres—mm), and mass (in grams—g), were taken for every photographed olive by using:

- a KERN PCB 3500-2 precision balance (KERN & Sohn GmbH, Balingen, Germany).
- a 0.01 mm-resolution 0.02 mm-accuracy Electronic Digital Vernier Caliper.

The values were annotated and associated to the position of the corresponding olive fruit in the image in which it appeared.

### 2.2. Image Analysis and Segmentation

The proposed methodology is aimed at automatically extracting from the images features descriptive of the mass and size of the olive fruits. To accomplish this task, the developed algorithm uses techniques based on mathematical morphology and segmentation by clustering-based image thresholding. This algorithm was implemented using MATLAB and Image Processing Toolbox Released 2016a (The MathWorks, Inc., Natick, MA, USA).

#### 2.2.1. Preprocessing

Firstly, images are down-scaled to 40% of its original size using bicubic interpolation for the decreasing of the computational workload. Next, a *salt-and-pepper* noise reduction is accomplished by applying a gaussian filter (rotationally symmetric gaussian low-pass filter) with a standard deviation of 0.8, and a kernel size of 5×5.

Secondly, images are transformed from the native RGB color space to HSV [21]. After studying the characteristics of the images, it was concluded that the RGB space did not offer an optimal data representation for the purposes of this study. In terms of color, an absence of homogeneity between the olive fruits was detected (especially for the Arbequina variety), which prevented it from being exploited as a distinctive feature. Conversely, the difference between the fruits and the white background in terms of lightness/brightness is remarkable. The level of lightness/darkness of the color of a pixel can be accessed by transforming its RGB values in accordance with a different representation of this color model. Notwithstanding this, basing the process exclusively on light intensity could not yield good segmentation results. Indeed, there were background pixels with lightness values similar to those of olives due to the shadows cast by these fruits. At this point, it was observed that color saturation also provided object differentiation while keeping similar values for background pixels, including both the ones which belonged to a shadow and the ones that did not. Nevertheless, despite this being a partial solution to the shadow problem, the segmentation based merely on saturation couldn’t yield reliable results, leading to olive pixels with saturation levels close to the background values which lacked accuracy. Therefore, neither color saturation nor intensity were found to be fully effective for image segmentation by themselves; however, an accurate combination and processing of both appeared more effective. Due to these reasons, HSV color space provided a solution, as it provides the saturation and value (level of lightness/darkness of the color) information separated into different layers (*S* and *V* channels, respectively). It is important to note that other existing color spaces are potentially valid according to this scenario, such as HSL [21] or CIELAB [22], among others.

#### 2.2.2. Image Segmentation

**Figure 2:**
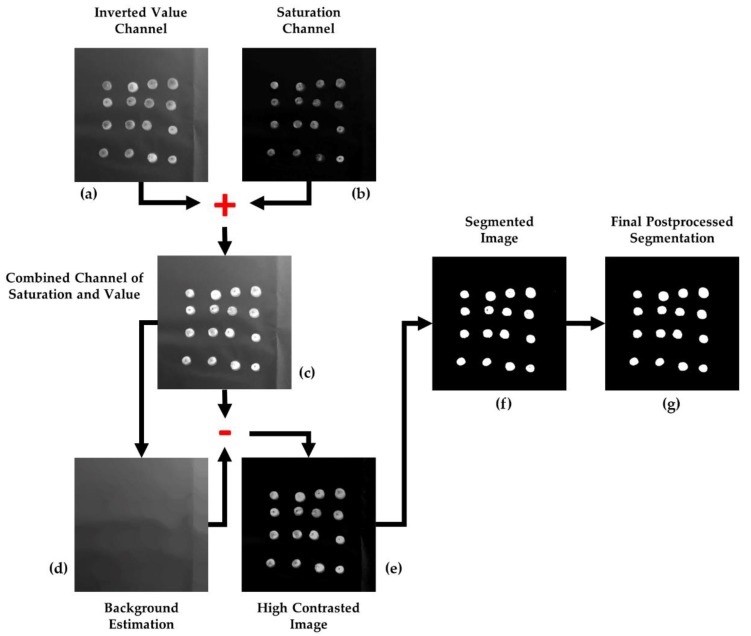
Step by step illustration of the image analysis algorithm on a sub-image of the study set: (**a**) inverted value channel; (**b**) saturation channel; (**c**) combined channel of saturation and value; (**d**) background estimation; (**e**) high contrasted image; (**f**) segmented image; (**g**) final postprocessed segmentation.

Once the image is transformed into the HSV color space, the value and saturation channels are isolated into different matrices, *V* and *S*, respectively. These matrices are transformed and combined into a unique component that it is treated as a grayscale image, which is the one to be segmented. According to this, as a first step, the elements of the *V* component are inverted with regard to the maximum possible grey-value, i.e., 255 (for 8-bit per channel image quantification). As such, given *V* is the image defined in the interval [0, 255], the image *V_INV_* is the one resulting from the next operation, as can be examined in Figure 2a:

(1) $$V_{INV}=255-V$$

Considering the *V* channel as a greyscale image, the aim of this transformation is to set the higher grey values to olive pixels and, consequently, the lower values to the background, which becomes the darkest part of the image. Then, the saturation layer (Figure 2b) is combined to the outcome of this transformation, as is shown in Figure 2c, looking forward to improving the contrast between the background and foreground and to complement information from both sources:

(2) $$I_{SV}=S+V_{INV}$$

Next, with the purpose of obtaining a background estimation, a morphological opening is applied to $I_{SV}$:

(3) $$I_{\gamma }=\gamma_{\beta }\left(I_{SV}\right)=\delta_{\beta }\left(\varepsilon_{\beta }\left(I_{SV}\right)\right),$$

where *β* is a 50-pixel-radius disk-shaped structuring element, large enough to contain any olive, and *δ* and *ε* are the basic morphological operations of dilation and erosion, respectively [23]. The result of this operation can be checked in Figure 2d. Then, the values of the background estimation are subtracted from $I_{SV}$, thus computing a high-contrast image:

(4) $$I_{HC}=I_{SV}-I_{\gamma }$$

The outcome of this operation, $I_{HC}$, is the grayscale image to be segmented by binarization. To automatically set an optimum global threshold, the clustering-based method proposed by *Otsu* [24] was selected. This method starts from the premise that the image contains two normal-like distributions of pixels, corresponding to the foreground and the background. Then, the threshold is decided as that which maximizes the inter-class- or minimizes the intra-class-variance to optimize separation. This approach explodes the characteristics of image $I_{HC}$, which is the result of an image processing aimed at strengthening contrast between the olives and the background, and at homogenizing the latter to favor binarization using a global threshold. Therefore, by applying the Otsu’s method to $I_{HC}$, the threshold *thresh* is obtained and applied to undertake its binarization as:

(5) $$I_{BIN}\left(x,y\right)=\{\begin{array}{c} 255\;if\;I_{HC}\left(x,y\right)>thresh\\ 0\;in\;any\;other\;case \end{array},$$

**Figure 3:**
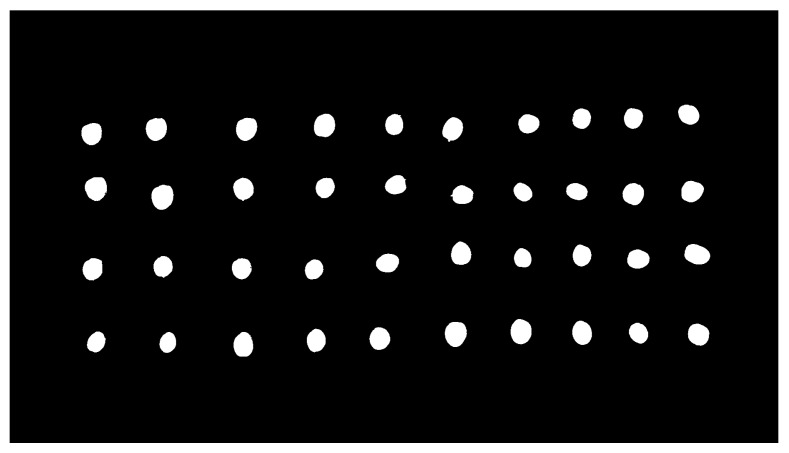
Result of the segmentation of the image of the A1 set, originally shown in Figure 1.

The result of the described methodology for olive fruit segmentation can be analyzed in Figure 2 and Figure 3.

#### 2.2.3. Postprocessing

As a last step, some morphological transformations are appealed to improve the final segmentation result. First, false positives filtering is addressed by eliminating those connected components that are too abnormally small to be considered as olive fruits. Mathematically:

(6) $$I_{BIN2}=\gamma_{\beta }\left(I_{BIN}\right),$$

where *γ* is the morphological opening with a disk-shaped structuring element *β* with a radius of 3 pixels.

Finally, a flood-fill operation is applied to eliminate false negatives represented by the small holes which have *emerged* inside some fruit-corresponding connected components (the holes derive from points of maximum reflection of light, because of the convex surface of the fruits).

(7) $$I_{DEF}=R_{I_{BIN2}}^{\varepsilon }\left(I_{m}\right),$$

where *R* is the morphological reconstruction operation, which consists on the iterative erosion (*ε*) of the image $I_{m}$ regarding to $I_{BIN2}$, using a unitary structuring element, until idempotence:

$$R_{I_{BIN2}}^{\varepsilon }\left(I_{m}\right)=\varepsilon_{I_{BIN2}}^{i}\left(I_{m}\right)$$

where

(8) $$I_{m}\left(x,y\right)=\{\begin{array}{c} I_{BIN2}\left(x,y\right),\;if\;\left(x,y\right)\;lies\;on\;the\;border\;of\;I_{BIN2}\\ \max\left(I_{BIN2}\right),\;otherwise \end{array},\;\mathrm{and}\;\varepsilon_{I_{BIN2}}^{i}\left(I_{m}\right)=\varepsilon_{I_{BIN2}}^{i+1}\left(I_{m}\right),\;\varepsilon_{I_{BIN2}}^{1}\left(I_{m}\right)=\varepsilon_{\beta =1}\left(I_{m}\right)\vee I_{BIN2}.$$

The corrective effect of this postprocessing is shown in Figure 2g.

### 2.3. Estimation Model Training

The goal here is to extract descriptive data from the segmented images to build estimation models for olive major and minor axis length, and mass. To this end, the binarized images allow us to work with the connected components representing the different olive fruits. First, to characterize the minor and major axis of the olives, for every component, the ellipse has the same normalized second central moments as it is being computed. Using this method, the major and minor olive axis are approximated to the major and minor axis of this ellipse, respectively, and their length in pixels is used for size estimation. On the other hand, the area of the segmented connected components, calculated as the number of constituent pixels (using 8-connectivity), is selected as a feature to estimate olive mass. 

Once this information is extracted, for each of the two considered varieties, a population of 50 individuals/olives is selected as the training set; the remaining 150 individuals are kept for external validation. These training sets are representative of the variability of the samples regarding the features under study. Next, the measurements of the major and minor axis length, and mass, corresponding to these populations and extracted automatically as specified above from the segmented images, are compared to the objective measurements taken in the laboratory. Thus, via regression analysis, linear estimation models for the targeted magnitudes and specific to each variety are yielded. Additionally, variety independent models for the magnitudes are also calculated by joining the two training sets from the two varieties and applying the same described procedure.

## 3. Results and Discussion

### 3.1. Evaluation of the Image Analysis Algorithm

**Figure 4:**
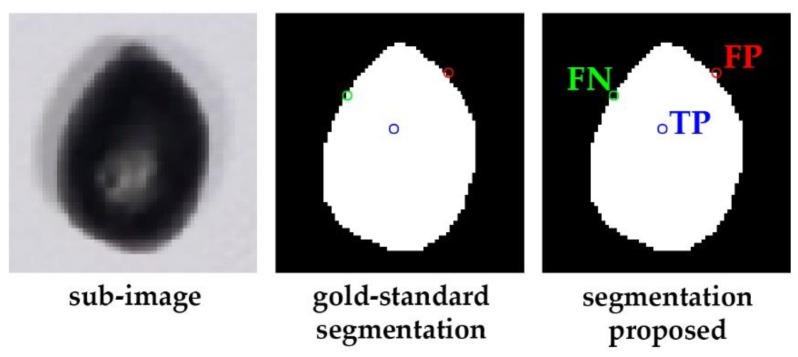
Examples of the different categories of pixels established to evaluate the segmentation results: *true positives* (TP, in blue); *false positives* (FP, in red); *false negatives* (FN, in green).

Every segmented image obtained with the proposed methodology is compared to a corresponding reference image at a pixel level to evaluate its quality. To enable this comparison, a ground-truth image was generated per each image-set considered in the experiment (A1–A5 and P1–P4) by manually labeling pixels using a graphic editor (concretely Photoshop CC V 14.0, Adobe Systems Incorporated, San Jose-California, EEUU); olive and background pixel values were set to 255 and 0, respectively. Then, results of pixel comparisons are categorized and annotated according to the following definitions (see Figure 4 to check each case):

- TP: Those foreground/olive pixels in the segmented image (white pixels) matching with their analogue ones in the corresponding ground-truth image (they keep being white pixels).
- FP: Those foreground/olive pixels in the segmented image (white pixels) that were labeled as background (black pixels) in the corresponding ground-truth image.
- FN: Those background pixels in the segmented image (white pixels) that were labeled as foreground/olive (white pixels) in the corresponding ground-truth image.

Thereby, segmentation quality can be finally assessed using the widely used Precision (*PC*) and Recall (*RC*) metrics, which are formulated as:

(9) $$PC=\frac{TP}{TP+FP}$$

(10) $$RC=\frac{TP}{TP+FN}$$

Thus, *PC* calculates the rate of correctly-detected olive pixels, and *RC* gives the rate of the actual olive. Finally, as a metric combining both *PC* and *RC* to provide with an overall accuracy measure of the segmentation method, *F*-score was calculated using the next common definition:

(11) $$F=2\frac{PR\times RC}{PR+RC}$$

### 3.2. Results of the Image Analysis Algorithm

As was mentioned previously, the validity of the image-segmentation algorithm has been tested through ground-truth image comparison. Based on the measures proposed to evaluate the algorithm performance, the yielded results are shown in Table 1.

Generally speaking, there are no outstanding differences between the two varieties in terms of algorithm performance. This fact suggests the method’s viability as a variety-independent method, and it supports the initial decision of not basing it on color features (due to hue usually being a differential distinctive feature among varieties).

Interestingly, it is noticeable that high PC values were obtained, despite the lack of uniformity of the background (due to the folds of the plastic mat, as can be observed in Figure 1). This lack of uniformity implies more noise, which could provoke the increase of false positive pixels (FP), and, consequently, the impoverishment of the results in terms of precision. To avoid this phenomenon, the estimation and subtraction of the background are performed. For the hypothetical implementation of the presented method in an actual system, other materials could be explored in order to obtain a more homogeneous background, thus favoring the method’s reliability. Nevertheless, since it can’t be expected in an ideal scenario, background estimation/subtraction must be considered as a key part of the method. 

On the other hand, it is important to underscore that the number of connected components isolated by the methodology was exactly matched with the number of olive fruits in all the images. This result is especially remarkable when considering a future commercial application of the presented solution for counting olives in a processing chain.

### 3.3. Evaluation of the Estimation Models

To evaluate the quality of the estimations produced by the different estimation models on the external valuation sets, the following metrics are proposed:

- Root-Mean-Square Error:
  (12) $$RMSE=\sqrt{\frac{\sum_{i=1}^{n}{\left(\hat{y_{i}}-y_{i}\right)}^{2}}{n}}$$
- Relative Root-Mean-Square Error expressed as percentage
  (13) $$SE=\frac{RMSE}{\frac{\sum_{i=1}^{n}y_{i}}{n}}\times 100$$
- Relative Mean Error expressed as percentage
  (14) $$\left|E\right|=\frac{\left|\sum_{i=1}^{n}\left(\hat{y_{i}}-y_{i}\right)\right|}{\sum_{i=1}^{n}y_{i}}\times 100$$
  where, for a feature under study (major axis, minor axis or pixel-area/mass), $\hat{y_{i}}$ is the predicted value and $y_{i}$ is the actual value (measured previously in the laboratory), for the *i*-th olive-fruit processed; *n* is the total number of olive fruits considered.

In addition, one-way analysis of variance was addressed on the estimation results of the different developed models for the two varieties. Mean comparison was attempted, using the Tukey’s test [25] at p<0.05, on the population of individual relative errors defined as the ratio between the estimated and the actual value considered:

(15) $$e_{i}=\frac{\hat{y_{i}}}{y_{i}},$$

where $\hat{y_{i}}$ and $y_{i}$ has the same meaning as defined above.

### 3.4. Results of the Estimation of Olive Features

**Figure 5:**
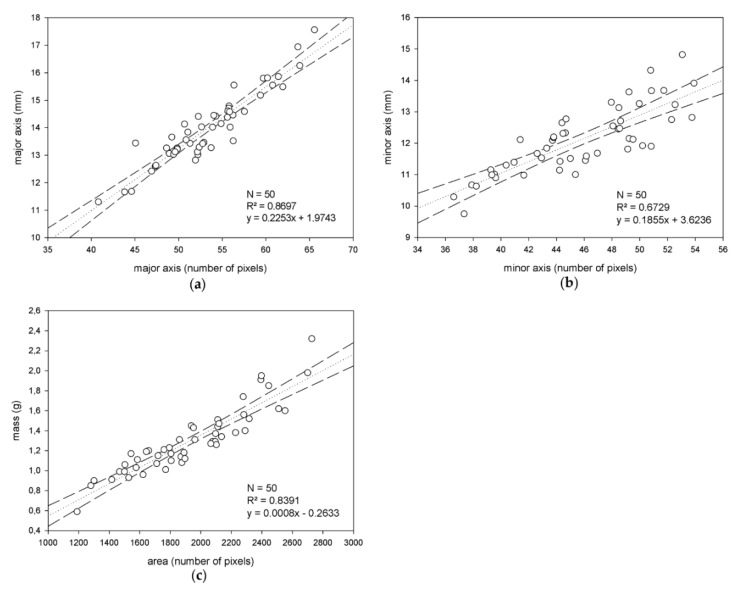
Correlation study performed for the Arbequina variety, considering the three different sizing features of the fruits the experiment is focused on: The major axis (**a**), minor axis (**b**) and mass (**c**).

**Figure 6:**
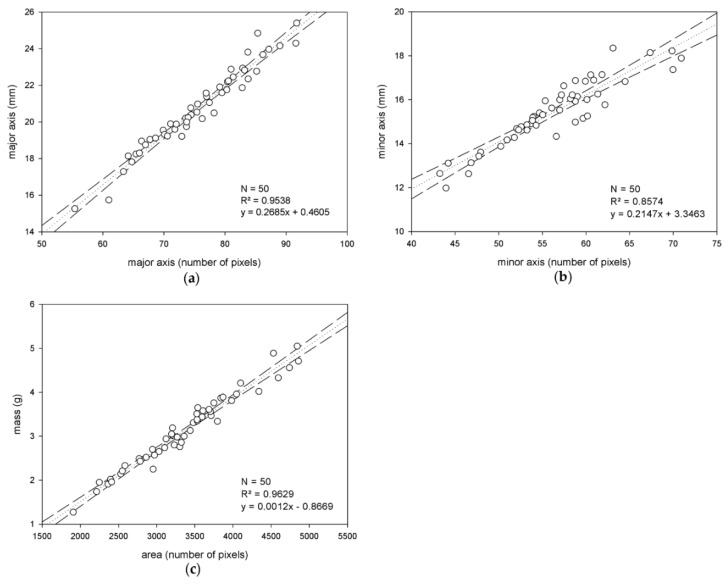
Correlation study performed for the Picual variety, considering the three different sizing features of the fruits the experiment is focused on: The major axis (**a**), minor axis (**b**) and mass (**c**).

First, the results of the correlation study performed on the training sets to obtain the estimation models are analyzed here. Separately for the training sets of Arbequina and Picual, the correlations found between the series of data pairs, ‘magnitude measured in image’ vs. ‘actual magnitude measured in the laboratory’, for the features under study, are illustrated in Figure 5 and Figure 6.

Globally, there was a positive correlation in all cases, which indicated promising estimation perspectives on the validation sets for all of the features. Nevertheless, there are a few considerations to highlight. First, despite the similar segmentation quality outcomes (Table 1), the correlation results were noticeably better for the Picual variety. Regarding mass modeling, this behavior may be explained by the fact that the method approaches the problem using the projection of the connected components representing the olives onto a 2D plane. Indeed, in that projection, every pixel has the same contribution to the mass of the fruit. This lineal approximation, despite being potentially valid attending to the correlations obtained (to be confirmed later with the validation results), may benefit some varieties more than others depending on their morphological characteristics. Second, to analyze the training correlation results corresponding to the diameters of olives, it is important to underscore that some caution is advised when taking the objective measurements with the digital caliper. Indeed, the lack of firmness of the fruits inevitably induced certain variability in caliper jaws fitting. Since firmness is a defining characteristic of olive fruit varieties, the impact of this variability could be different depending on the kind of fruits under study. This fact has to be considered when assessing the results, and it could partially explain the best correlation for Picual compared to that for Arbequina in terms of size features. On the other hand, the visual determination of the minor axis was found to be more non-specific than for the case of the major axis. Thereby, it also introduced a new variability factor to consider which may explain, at least partially, the correlation differences between the two axes, thus not being univocally imputable to the developments presented here.

**Figure 7:**
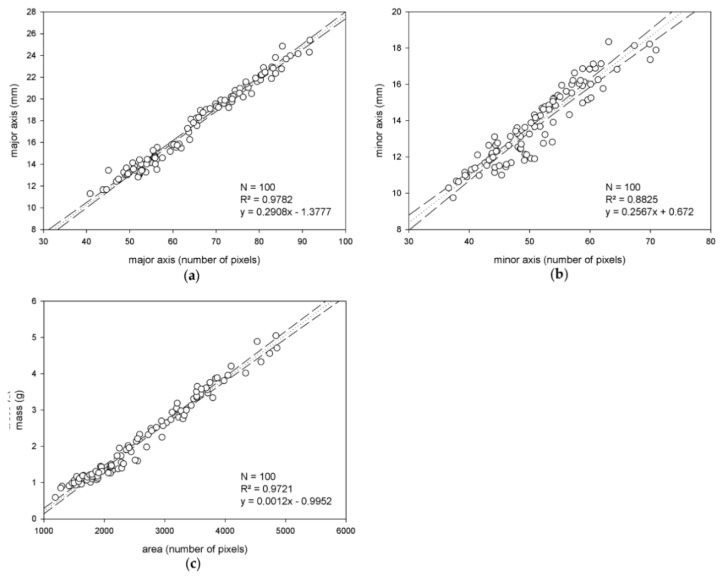
Correlation study of variety-independent model trained on the instances from both Arbequina and Picual varieties, considering the three different targeted features: The major axis (**a**), minor axis (**b**) and mass (**c**).

The previous approach comprised the development of models specific to each variety. To explore the idea of variety-independent modeling, the two training sets were configured as a unique population to be correlated, thus producing functions applicable to estimate the features of both cultivars. The scatter plots shown in Figure 7 illustrate the training results, which resulted in even higher correlation values than those obtained in the previous case. This could be explained because of a wider domain of the values of the targeted features, which could provide a better adjustment.

To evaluate the quality of the different estimation models, the measures proposed before to quantify the differences between the predicted values and the observed ones, have been calculated after applying the models on the corresponding external validation sets. The results can be found below, in Table 2.

As can be analyzed, the results produced by the variety-independent models are comparable to those given by the functions specifically trained for each cultivar. This indicates that there is no clear evidence of benefits in the use of specific models to the detriment of the more general solution. This fact increases the expectations on generality and usability of the proposed method. Moreover, the numerical results (with relative mean errors (|E|) below 2.5% for all cases; Equation (14)) do not clearly support the need for exploring non-linear solutions, that are far more complex in order to handle and be more sensitive to training populations.

With the aim of determining the probability of getting performance quality for each of the diverse varieties, a one-way analysis of variance was accomplished. To achieve this, the individual relative errors (Equation (15)) produced by the models, for the three features, on the samples in the external validation sets of Arbequina and Picual, were calculated. Then, statistical differences between the populations were studied by mean comparison using the Tukey’s test [25] at p<0.05. Table 3 shows the results of this analysis for the two modeling approaches and the three features. In the case of the specific prediction models, no statistical differences were found between Arbequina and Picual for the estimations of the minor axis. Conversely, significant differences were found for the estimations of the major axis and mass. On the other hand, the analysis concluded complementary results for the estimations produced by the variety-independent models. This is, statistically significant differences were found for the minor axis estimations, whilst the major axis and mass estimations verified the null hypothesis. These outcomes reinforce the previous discussion about the suitability of the variety-independent solution.

## 4. Conclusions

In the present paper, a method based on image analysis techniques has been developed for estimating the size and mass of olive fruits. The results underscore the robustness and accuracy of the algorithm this method is based on. Moreover, they support its viability for the development of sorting and grading systems for the olive industry.

In accordance with the results, the segmentation algorithm showed a noticeably good performance in the image segmentation binarization task when compared to ground-truth images. Additionally, it was able to detect the exact number of fruits that appeared in every treated image, thus highlighting the accuracy of the process. It is also interesting to note the steadiness of the method dealing with two different olive fruit varieties, as this increases confidence in its applicability to other cultivars. Nevertheless, future trials will focus on analyzing this aspect of the method to verify this generality. Also, these trials could explore different lightning systems, such as diffuse illumination, which could improve the image acquisition task by minimizing the shadows cast by the fruits, thus enabling more reliable segmentation results.

Regarding the estimation of the major and minor axis, and mass of olives, accurate results were measured, which do not indicate the necessity of exploring non-linear modeling to this effect. Especially remarkable is the analyzed behavior of the variety-independent models, which showed comparable, or even better, performance than specific models. This outcome supports their use in the pursuit of applicability and generalization. Notwithstanding this, future investigations will pursue the verification of this conclusion with studies that include samples from more varieties. Moreover, further and wider investigations will also be conducted to more confidently quantify the impact of pixel weighing linearization for mass estimation.

On the other hand, there is a requirement for the proposed methodology to be applied, in terms of the disposition with the olive fruits that are placed on the images. Thus, it is necessary that a certain minimum distance be maintained between every pair of fruits. This fact does not imply a problem in a real scenario, where a non-flat belt conveyor equipped with cleavages can be used, which provides a way to keep the fruits separated from each other. Nevertheless, further work might explore the enhancement of the image-binarization method presented, with the purpose of making possible a reliable segmentation that will correctly work in a scenario in which olives appear to be touching each other. Notwithstanding, it would probably require a considerable increase in algorithm complexity, so it remains to be determined if it could satisfy the working conditions of a real-time system.

The presented solution comprises a promising starting point to develop sorting and grading technologies based on image analysis, which would provide high value for the olive-manufacturing industry.

## Figures and Tables

**Table 1:** Performance of the segmentation algorithm calculated by comparison between the binary images automatically produced and the corresponding ground truths. Results are expressed in terms of Recall (RC), Precision (PC) and F-score, and detailed per variety, subset, and considering all the samples as a whole.

**Set/Image**	**RC**	**PC**	**F-Score**
**Arbequina**			
A1	0.9614	0.9372	0.9491
A2	0.9545	0.9432	0.9488
A3	0.9551	0.9535	0.9543
A4	0.9536	0.9745	0.9639
A5	0.9510	0.9510	0.9510
Overall	0.9551	0.9519	0.9534
**Picual**			
P1	0.9464	0.9810	0.9634
P2	0.9414	0.9922	0.9661
P3	0.9380	0.9869	0.9618
P4	0.9316	0.9967	0.9631
Overall	0.9393	0.9892	0.9636
**Arbequina + Picual**			
Overall	0.9481	0.9685	0.9580

**Table 2:** Estimation results, calculated on the external validation sets, for the three studied features detailed per variety and modeling approach. Outcomes are expressed in terms of Root-Mean-Square Error (RMSE; Equation (12)), Relative Root-Mean-Square Error (SE; Equation (13)), and Relative Mean Error (|E|; Equation (14)).

**Arbequina Validation Set (N = 150)**				
**Feature**	**Estimation Model**	**RMSE**	**SE (%)**	|E| **(%)**
Major axis	Specific	0.4885 (mm)	3.46	0.86
	Variety-independent	0.5778 (mm)	4.09	0.14
Minor axis	Specific	0.6007 (mm)	4.99	0.09
	Variety-independent	0.7811 (mm)	6.49	2.39
Mass	Specific	0.1220 (g)	9.62	0.78
	Variety-independent	0.1775 (g)	13.99	1.51
**Picual Validation Set (N = 150)**				
**Feature**	**Estimation Model**	**RMSE**	**SE (%)**	|E| **(%)**
Major axis	Specific	0.4163 (mm)	1.98	0.03
	Variety-independent	0.4770 (mm)	2.27	0.60
Minor axis	Specific	0.6804 (mm)	4.38	0.29
	Variety-independent	0.8036 (mm)	5.17	1.53
Mass	Specific	0.250 (g)	7.89	2.39
	Variety-independent	0.2439 (g)	7.69	1.65

**Table 3:** Results of one-way analysis of variance performed on the Arbequina and Picual estimations produced by the specific and variety-independent models. The analyzed populations are the individual relative errors (Equation (15)) produced by the different models on the external validation sets of each variety. The mean ($\bar{X}$) and standard deviation (*σ*) of each population is given. Dissimilar letters indicate different statistical means according to the analysis of variance using the Tukey’s test [25] at p<0.05.

**Specific Estimation Models (N = 150)**		
**Feature**	**Arbequina** $\left(\bar{X},\;\sigma \right)$	**Picual** $\left(\bar{X},\;\sigma \right)$
Major axis	(0.9921, 0.0344) ^a^	(1.0005, 0.0199) ^a’^
Minor axis	(1.0026, 0.0502) ^b^	(1.004, 0.043) ^b^
Mass	(0.9985, 0.0946) ^c^	(1.0275, 0.0741) ^c’^
**Variety-Independent Estimation Models (N = 150)**		
**Feature**	**Arbequina** $\left(\bar{X},\;\sigma \right)$	**Picual** $\left(\bar{X},\;\sigma \right)$
Major axis	(1.0011, 0.042) ^a^	(0.9936, 0.0221) ^a^
Minor axis	(1.0246, 0.0611) ^b^	(0.9847, 0.0486) ^b’^
Mass	(1.0068, 0.1428) ^c^	(0.9851, 0.0731) ^c^
